# Effect of community based practice of Baduanjin on self-efficacy of adults with cardiovascular diseases

**DOI:** 10.1371/journal.pone.0200246

**Published:** 2018-07-30

**Authors:** Xiangli Xiao, Jian Wang, Yanmei Gu, Yanfang Cai, Lixin Ma

**Affiliations:** 1 Nursing Department, Hebei Provincial Children's Hospital, Shijiazhuang, Hebei, China; 2 School of Nursing, HeBei University of Chinese Medicine, Shijiazhuang, Hebei, China; 3 Health Service Center of XiLi Community, Shijiazhuang, Hebei, China; New England School of Acupuncture, UNITED STATES

## Abstract

**Background:**

Low self-efficacy in chronic disease patients is one of the main disturbances which require physical and mental rehabilitation, calling for the development of a home accessible way to improve self-management.

**Objectives:**

The purpose of this study was to explore the effectiveness of a community based Baduanjin exercise on self-efficacy in adults with cardiovascular disease.

**Design:**

A randomized controlled trial, longitudinal research design was employed.

**Participants:**

After screening by health documents in Community Health Service Station, a total of 134 patients with records of cardiovascular diseases were had been enrolled according to the following inclusion criteria: (1) Community dwelling adults in Xili Community; (2) Patients diagnosed with cardiovascular diseases by community doctors, or other clinicians in health records in the past 3 years (2013–2015); (3) independent walking.

Participants were excluded if they: (1) had impaired mobility and limited extremities functionality; (2) had not been in stable health condition and could not adhere to the exercise regime; (3) had communication difficulties and limited ability to follow instructions.

**Methods:**

Participants were randomly assigned to the Baduanjin group or the control group. Those in the Baduanjin group received 16 weeks of Baduanjin exercise training, while those in the control group kept the original exercise mode unchanged. The Self-Efficacy for Managing Chronic Disease 6-item Scale (SEMCD6) was administered to subjects before and after intervention.

**Results:**

Demographic data showed that 65.12% of the enrolled 129 participants were aged 65 or older, 92.25% received less than 12 years of education, and 68.21% participants’ monthly income was less than 1999 RMB. Before intervention, SEMCD6 scores of 86.36% participants in Baduanjin group were below 7 points, while 85.71% in control group; after 16 weeks of Baduanjin exercise, SEMCD6 scores lower than 7 points in Baduanjin group (21.21%) were significantly lower than that of the control group (84.13%). The increase of SEMCD6 scores in Baduanjin group was statistically significant in the confidence to keep the fatigue, to keep the physical discomfort or pain, to keep the emotional distress and do the different tasks and activities (*P*<0.01).

**Conclusions:**

Adults with cardiovascular diseases in community have lower level of education, most of whom have a low monthly income; thus, community dwelling cardiovascular disease patients are more suitable for an economic program to persist their long term management of the disease. Baduanjin is a traditional Chinese medicine regimen with less physical and cognitive demand; community based exercise of Baduanjin could help to increase self-efficacy in patients with cardiovascular diseases, thus better self-management of rehabilitation process.

## Introduction

Cardiovascular disease (CVD) is one of the four non-communicable diseases that threaten the health of human beings [1]. China is a country with high and fast increasing incidence of CVDs with around 290 million patients. CVDs have become the leading cause of mortality in both urban (42.61%) and rural (45.01%) area according to the annual CVD report [2]. Physical inactivity is listed as one of the major risk factors to CVDs. National-level data show that from 1991 to 2011, physical activity level in adults (18 to 60 years old) markedly decreased in China [2].

Change of sedentary life style to prevent CVDs requires adherence to a routine physical exercise practice. Regular exercise is frequently recommended by doctors for CVDs patients, but poorly obeyed. It is the patients who decide what they will do every day. Nevertheless, each individual is facing different social and physical environment. They need confidence to deal with the complex situation and manage the health problem. Bodenheimer states that internal motivation as more effective for life style change than external motivation [3]. In practice, self-efficacy to perform physical exercise is more important than the physician’s prescription itself and plays a crucial role to better adherence. Self‑efficacy is known as “the perceived confidence in the ability to take successfully action and perform a specific task.” It is a prerequisite of effective self-management[4]. Measured by a 1 to 10 scale, if the patients rank the confidence higher than 7, the action plan is likely to be accomplished [5]. Thus, the health education should focus on patients’ coping skills and confidence to manage health problems. Cross-section studies show that self-efficacy for managing chronic disease (SEMCD) is one of the essential elements in successful self-management of the diseases and behavior change [6,7,8,9,10]. However, insufficient data are available targeted on self-efficacy in patients with chronic diseases, though some positive results have been achieved in improving health outcomes.

Traditional Chinese medicine ascribes great importance to the prevention and treatment of CVDs. Baduanjin is a widespread traditional fitness Qigong in China, which possibly dates back to Song Dynasty (960–1279)[11]. It is recommended by the Chinese Qigong Association for health promotion. As one of the simplest and least physical demanding Qigong exercise, it is widely practiced by people at all ages [12,13,14,15,16]. Baduanjin exercise involves body posture and coordinates movement with meditation and breathing [11]. Studies have demonstrated physiological benefits of Baduanjin and suggest that regular practice of Baduanjin results in significant improvement in cardiopulmonary function [16,17] and sleep quality [13,14]. Furthermore, as a mind-body exercise, Baduanjin has also shown positive psychological effects for sub-health and mental problems by improving cognitive function [18,19,20]. Therefore, Baduanjin practice is associated with not only physical outcomes, but also improvement in one’s confidence to cope with negative emotions [16,21]. Based on these findings, we speculated that Baduanjin might have positive effects on self-efficacy in patients with chronic CVDs. The purpose of the current study was to exam the effect of a community based Baduanjin exercise on self-efficacy in adults with CVDs.

## Methods

### Design

A randomized controlled trial, longitudinal research design was employed.

### Ethical approval

The protocol for this study was approved by Ethic Committee of Hebei University of Chinese Medicine.

### Setting and participants

**Fig 1:**
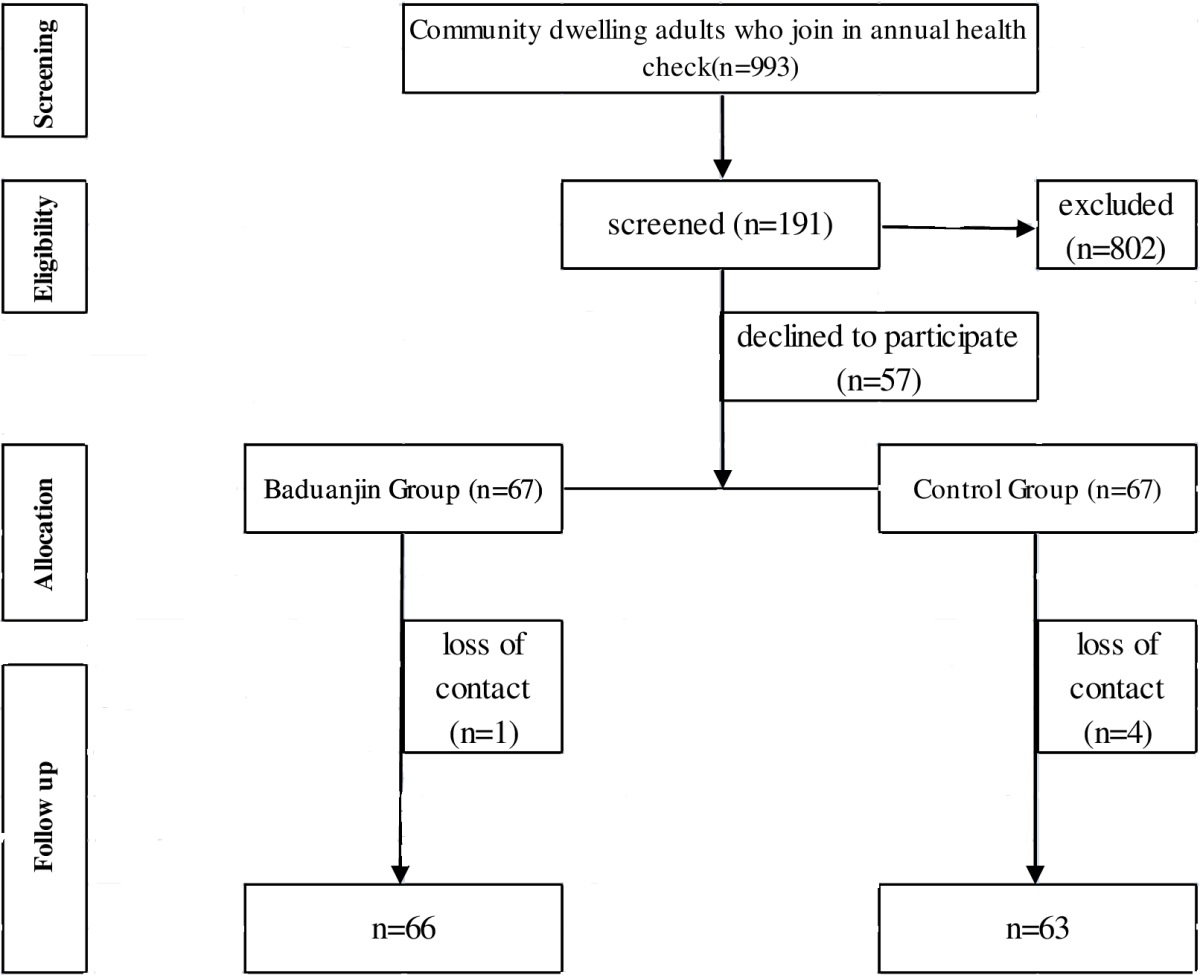
Flow of participants.

Community dwelling adults who take annual health check and have record achieves in Community Health Service Station were selected as research subjects. Screened by information from the health records in the past 3 years (2013–2015), subjects who met the following inclusion criteria were invited with phone calls: (1) community dwelling adults in Xili Community; (2) diagnosed with CVDs by community doctors, hospital doctors or other clinicians in health records in the past 3 years (2013–2015); (3) independent walking. Participants were excluded if they: (1) had impaired mobility; (2) not stable in health condition and could not adhere to the exercise; (3) had communication difficulties. Volunteers were recruited and trained in community center in Xili community in our city from March to June 2016 (Fig 1). Research purposes and methods were explained by project manager. A written informed consent was obtained before the intervention which was signed by each participant. They were assured of confidentiality and the option to withdraw at any time without penalty. After 16 weeks of intervention, all adults, with or without CVDs or other health conditions joined in the community based exercise at their own willingness, which was not displayed in Fig 1. The individual in this manuscript has given written informed consent (as outlined in PLOS consent form) to publish these case details.

### Intervention

**Fig 2:**
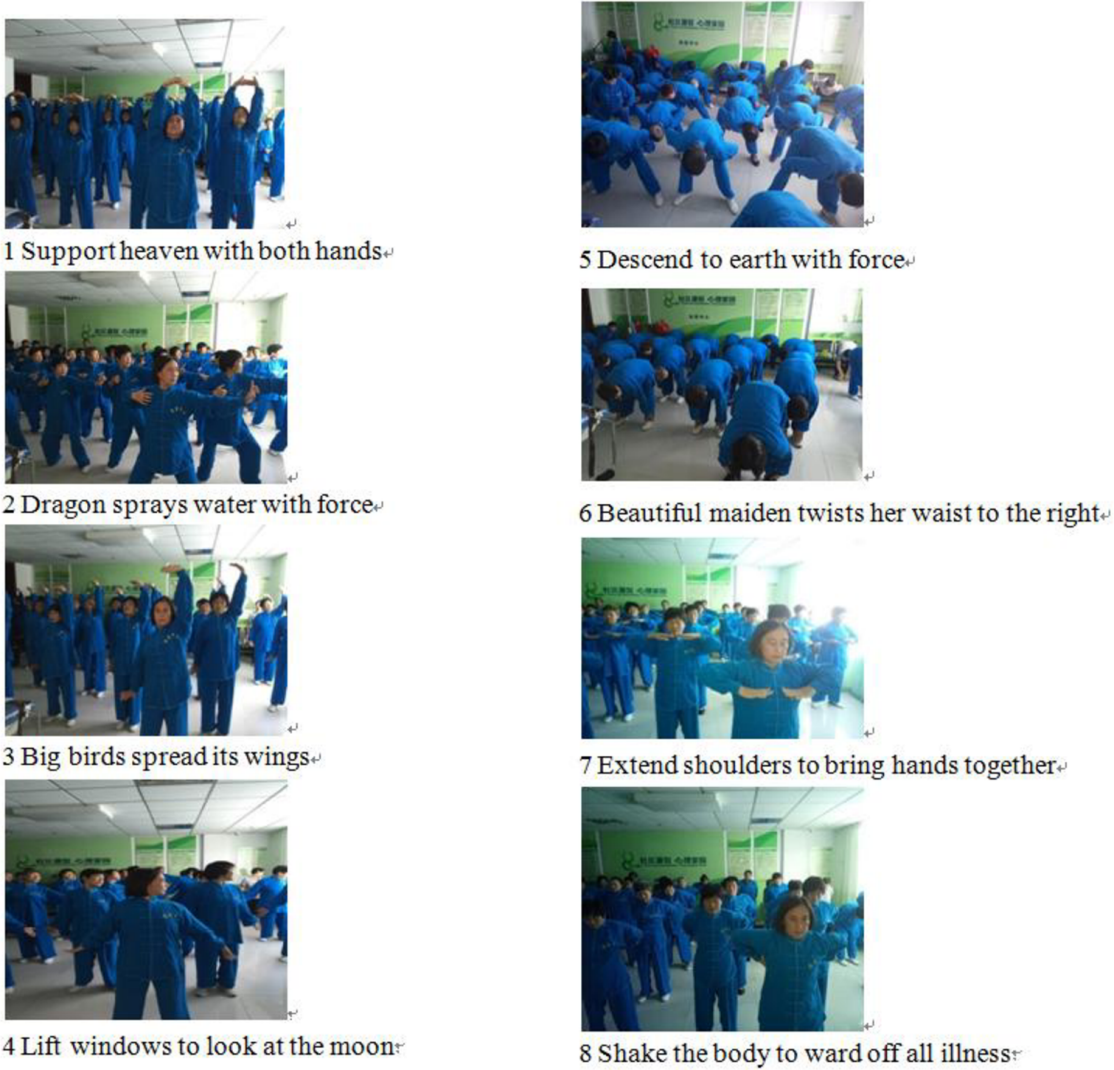
Community based Baduanjin exercise.

A total of 134 participants were randomly assigned into either the Baduanjin exercise group or the control group. Subjects in Baduanjin group completed a supervised Baduanjin exercise program for about 24 min each time, 5 times a week for 16 weeks. Following the video of *Fitness Qigong Baduanjin*, compiled by the Health Qigong Management Center of the National Sports, participants practiced with two supervisors to correct their movement and gestures (Fig 2). Accuracy of practice was assessed through observations by researchers for each time. Participants were encouraged to attend next practice by peer encouragement and by supervisors. On the other hand, participants in control group were not given any interventions but instructed to continue their usual activities.

### Measurements

#### Demographics and clinical characteristics

Information about gender, age, marital status, education level, occupation, family monthly income, living arrangement, co-morbidities, smoking, medication and regular exercise were obtained before intervention by a self-administered questionnaire.

#### Self-efficacy measurement

Self-Efficacy for Managing Chronic Disease 6-item Scale (SEMCD6) was used to assess self-efficacy before and after intervention [10]. SEMCD6 contains 6 items with a 10 step Likert scale ranged from 1 “not at all confident” to 10 “totally confident.” The level of confidence to fatigue, pain, the emotional distress, any other control symptoms, and actions to control illness were tested. The internal consistency coefficient is reported 0.77–0.92, and the test-retest reliability is 0.72–0.89. Scores range from 1 to 10 with higher scores indicating better self-efficacy.

#### Data analysis

SPSS19.0 package was employed to statistic description and analysis. Change in SEMCD6 scores were the main outcome of this study. The proportion of scores less than 7 was reported and emphasized. Chi-square tests were used to determine the demographic differences on baseline and to test whether there was difference of score increase (before vs. after intervention) between the two groups. Paired *t*-test was used to test score difference before vs. after intervention in Baduanjin group. An alpha level of *P* < 0.05 was considered significant for all statistical tests.

## Results

### Participants’ characteristics

Demographic data show that 65.12% (84/129) of the observed 129 participants were aged 65 or older, 92.25% (119/129) received less than 12 years of education, 68.21%(88/129) participants’ monthly income were less than 1999 RMB. We noticed that 7 participants (3 in Baduanjin group and 4 in control group) did not report medication intake though diagnosed as CVDs. There is a higher female proportion in the participants (male: female = 28:38 in Baduanjin group, and 22:41 in control group).

### Baseline comparison of subjects’ characteristics between two groups

Table 1 shows that distribution of the demographic data between Baduanjin group and control group has no statistical differences (p = 0.192–0.904). Table 2 shows proportion of participants with SEMCD6 scores lower than 7 before intervention in both groups. Chi-square test shows no statistical difference (χ2 = 0.011, p = 0.915).

**Table 1:** Baseline characteristics between Baduanjin and control groups.

**Characteristics**		**Baduanjin group**	**Control group**	**χ**^**2**^	***p***
**Total included in the final analysis**		66	63		
**Gender**	Male	28	22	0.765	0.382
	Female	38	41		
**Age–yr**	≤64	24	21	0.130	0.718
	≥65	42	42		
**Marital status**	Married	54	48	0.675	0.714
	Other status	12	15		
**Education–yr**	≤12	62	57	1.590	0.811
	≥13	4	6		
**Occupation**	Manual worker	39	39	1.573	0.904
	Office worker	3	3		
	Unemployed/retired	24	21		
**Family monthly income** (RMB)	≤1999	42	46	3.204	0.361
	≥2000	24	17		
**Living arrangement**	Alone	5	7	1.315	0.726
	With others	61	56		
**Co morbidities**	YES	39	34	0.344	0.557
	NO	27	29		
**Smoking**	YES	14	11	0.290	0.590
	NO	52	52		
**Taking medication**	YES	63	59	0.021	0.883
	NO	3	4		

**Table 2:** Comparison of low score (<7) on SEMCD6 between two groups.

Group	Before Intervention	After Intervention
Baduanjin group (66)	86.36%(57)	21.21%(14)
Control group (63)	85.71%(54)	84.13%(53)
***χ*^*2*^**	0.011	51.111
***P***	0.915	<0.001

### SEMCD6 score change before vs. after intervention

Table 3 demonstrates the improvement of SEMCD6 score after vs. before intervention in Baduanjin Group. In general, the total scores shows a significant increase from 6.52±0.54 to 6.92±0.38 (*P*<0.01).

**Table 3:** SEMCD6 score before e vs. after intervention in Baduanjin group (x±δ).

Items of SEMCD6	Before Intervention	After Intervention	*t*	*P*
Confidence to manage fatigue	6.33±1.40	6.98±1.02	-3.066	0.003
Confidence to manage physical discomfort or pain	6.32±0.99	6.65±0.64	-2.285	0.024
Confidence to manage emotional distress	6.56±0.99	6.95±0.75	-2.566	0.011
Confidence to manage symptoms	6.52±0.55	6.85±0.68	-3.031	0.003
Confidence to manage different tasks and activities	6.98±0.73	7.30±0.63	-2.643	0.009
Confidence to reduce influence of illness	6.44±0.89	6.82±0.78	-2.613	0.010
Total score SEMCD6	6.52±0.54	6.92±0.38	-4.893	0.000

**Fig 3:**
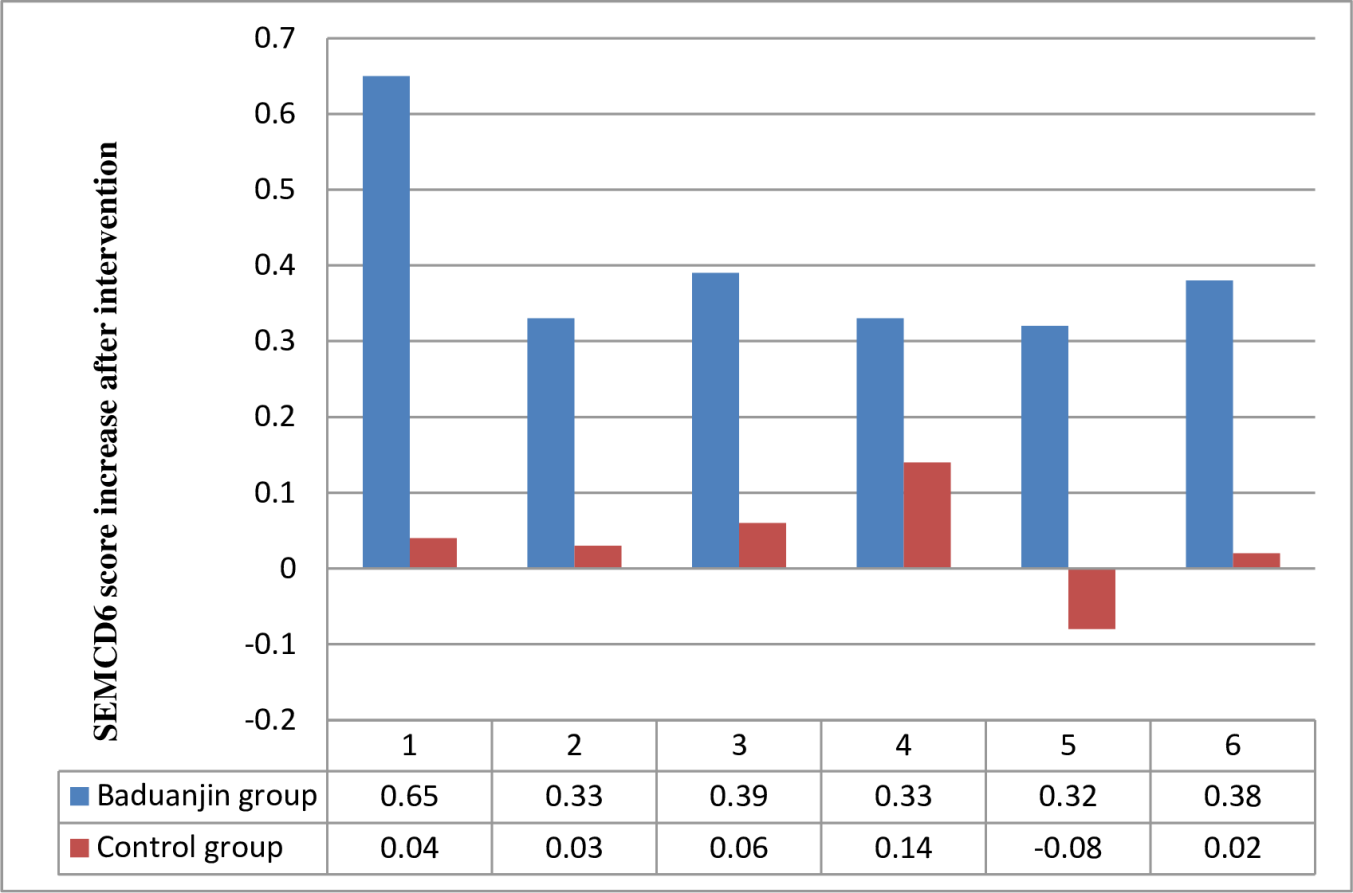
Comparison of average SEMCD6 score increase Notes for Fig 3: 1 How confident do you feel that you can keep the fatigue caused by your disease from interfering with the things you want to do? 2 How confident do you feel that you can keep the physical discomfort or pain of your disease from interfering with the things you want to do? 3 How confident do you feel that you can keep the emotional distress caused by your disease from interfering with the things you want to do? 4 How confident do you feel that you can keep any other symptoms or health problems you have from interfering with the things you want to do? 5 How confident do you feel that you can do the different tasks and activities needed to manage your health condition so as to reduce your need to see a doctor? 6 How confident do you feel that you can do things other than just taking medication to reduce how much your illness affects your everyday life?

Fig 3 shows comparison of SEMCD6 score change between the two groups. It shows that, compared with the control group, the average score changes of items 1 to 3 and 5 in Baduanjin group were with statistical significance (P<0.01).

### Comparison of low score (<7) on SEMCD6 between two groups before vs. after intervention

Table 2 shows that before intervention, the two groups had 86.36% (Baduanjin group) and 85.71% (control group) of patients with SEMCD6 score below 7 points; After intervention, the number of participants with low score decreased to 14(21.21%) in Baduanjin group.

## Discussion

### Characteristics of community participants

The subjects of our study were from community dwelling adults in a middle city in north China. According to the records of Community Health Service Station’s achieves, 993 people were diagnosed CVDs by community doctors or clinicians from other health care settings. It is worth mentioning that there was a higher female proportion in the participants. One reason for that is, in China, the retire age is 60 years old in male and 55 in female. Those, who have not yet retired, mostly have their health records in the companies. This is also why 65.12% of the observed 129 participants were aged 65 or older, which is much higher than the proportion in general population. On the other hand, generally, female residents were more willing to participate in a health check than male residence. Therefore, in our study, community dwelling adults with CVDs were mostly retired residents in need of health guidance. Meanwhile, in our study, 79.1% of the observed subjects had less than 12 years of education, and 68.21% participants’ monthly income were less than 1999 RMB. The distribution of age, occupation, education, gender and income characteristic in our current study is consistent with the similar study in north China [7]. These might have clinical implication for long term caregivers in community health service, that an economic, home available and easy to learn exercise program is preferred.

### Effectiveness of Baduanjin practice on self-efficacy improvement

Health system in China is confronted with a rising prevalence of chronic diseases, thus, to improve patients’ self-management is regarded as an important component of nursing care [22]. Self-efficacy is considered as an early step in of behavior change in self-management [23,24,25]. In our study, after 16 weeks of Baduanjin practice, the total score of SEMCD6 increased significantly. Furthermore, compared to control group, average score changes on confidence to keep the fatigue, to keep the physical discomfort or pain, to keep the emotional distress and do the different tasks and activities in Baduanjin group showed significant increase. The score changes within the group and average score change comparison between the two groups showed that long-term practice of Baduanjin has a remarkable effect on the confidence of self-management. Generally speaking, people with high level of self-efficacy may have a better participation in a rehabilitation exercise [26,27,28]. In our study, during the 16 weeks of practice, there was only one participant lost contact. The high adherence may benefit from leadership from the researchers and the support from peers. The researchers were from Chinese traditional medicine university, who played as authority, and offered positive outcome expectancy, which contribute to build up confidence to initiate the practice. On the other hand, the role of peer support may indicate the importance of the partnership factor in long term rehabilitation [29]. Though Baduanjin and other fitness qigong have been listed in the rehabilitation programs by Association of Chinese CVDs Rehabilitation and Prevention, there is no clear reference to individual practice or community practice. Our study indicate that community based practice contribute to individual’s self-efficacy of chronic disease management. Further research might explore the advantage and disadvantage of different ways to practice, by comparing the individual with community practice.

The primary objective of this study was to determine the effect of Baduanjin practice on level of self-efficacy which was measured by SEMCD6. The results obtained from this study are consistent with the original hypnosis. However, our data showed that the average scores of SEMCD6 in Baduanjin group are still below 7. Therefore, we highlighted statistical analysis of the comparison of low score (<7) on SEMCD6 between two groups before vs. after intervention. The findings suggest that long term practice of Baduanjin is effective not only in increasing patients’ confidence in self-care, but also in reducing the proportion of patients with low confidence.

Researchers agree that scores over or equal 7 in self-efficacy scale indicate higher possibility of accomplishing a goal or task [5]. Data showed that before intervention, the two groups had 86.36% (Baduanjin group) and 85.71% (control group) of patients with SEMCD6 score below 7 points, which is consistent with the research results of the prevalence of low self-efficacy in CVDs patients [6,7,30]. After intervention, the number of participants with low score decreased to 14(21.21%) in Baduanjin group, which was significantly lower than that in the control group. According health behavior change studies, it indicates that most of the participants in Baduanjin group may continue the practice after the intervention [26,31][26,31].

There are several limitations of our study which should be mentioned. First, we recruited only adults with health medical records in Community Health Service Station. Therefore, our sample could not cover those who do not attend annual health check, such as the advanced aged people, adults who have their health records in their employers, and young adults who seldom take health check. So, the results of our limited sample may not be generalizable to whole populations. Second, we recruited adults who joined in the exercise program on their will. Generally speaking, these people may represent those with higher self-confidence and higher adherence. Therefore, further study on self-efficacy could sample from a more representative population. Third, we reported only one aspect of Baduanjin practice, but there may be more benefits of the exercises, such as influence on life quality, economic benefits, and other long term outcomes which need more prospective studies.

## Conclusions

In our study, community dwelling adults with CVDs have lower level of education, most of whom have a low monthly income and are more suitable for an economic program to improve self-management. Baduanjin is a traditional Chinese medicine regimen with less physical and cognitive demand; community based exercise of Baduanjin could help to increase self-efficacy in patients with CVDs, and reduce the number of patients with SEMCD6 score below 7 points.
